# Resilience, organizational support, and innovative behavior on nurses’ work engagement: a moderated mediation analysis

**DOI:** 10.3389/fpubh.2023.1309667

**Published:** 2023-12-19

**Authors:** Feiyang Zhou, Keyu Long, Haiyan Shen, Zixuan Yang, Tingting Yang, Lu Deng, Jie Zhang

**Affiliations:** ^1^Clinical Nursing Teaching and Research Section, The Second Xiangya Hospital of Central South University, Changsha, Hunan, China; ^2^Xiangya School of Nursing, Central South University, Changsha, Hunan, China; ^3^Operating room, The Second Xiangya Hospital of Central South University, Changsha, Hunan, China

**Keywords:** work engagement, resilience, organizational support, innovative behavior, nurse, nursing management

## Abstract

**Objectives:**

To investigate the status of nurses’ work engagement and the relationship among resilience, organizational support, and innovative behaviors.

**Methods:**

In this cross-sectional study, we investigated 496 nurses in Hunan, China, from July 2022 to December 2022. A descriptive statistical approach, Pearson’s correlation analysis and Hayes’ PROCESS Macro Models 4 and 14 were used to analyze the available data.

**Results:**

The level of work engagement among nurses was found to be moderate. Resilience positively predicted work engagement among nurses. Organizational support played a partially mediating role in the association between resilience and work engagement. Furthermore, innovative behavior played a moderating role in the association between adaptive resilience and work engagement.

**Conclusion:**

Based on the results, greater attention needs to be paid to nurses’ work engagement. A high level of resilience, organizational support, and innovative behavior may increase work engagement among nurses. Nursing leaders can take measures to increase work engagement among nurses by improving nurses’ resilience and organizational support, and cultivating innovative behavior.

## 1 Introduction

The global nursing workforce shortage is well-known (1). According to the World Health Organization (WHO), there is a shortage of 7.2 million healthcare workers in terms of health needs, while the report of the Third Global Forum on Human Resources for Health estimates that the nursing gap will reach 12.9 million by 2035 (2). This is exacerbated by an aging population and the increasing burden of chronic caregiving (3, 4). Improving the efficiency of nursing staff and increasing the work engagement of nurses are proven ways to improve the current stressful situation.

Work engagement is a state of motivation and fulfillment characterized by high mental and physical energy levels, enthusiasm, dedication, and complete absorption in work activities (5). Previous studies have shown that work engagement is associated with work resources (6), leadership (7), and social recognition (8). A cohort study showed a significant positive association between work engagement and job performance (9). Rising healthcare costs, a global shortage of nurses, and the reality of the demand for quality care pose significant challenges to health systems and governments around the world (10). Work engagement in nursing is a focused, engaging, and dynamic nursing practice that stems from an environment of autonomy and trust and leads to safer, more cost-effective patient outcomes (11). Nurses account for 59% of all health professionals worldwide, and their work engagement plays an important role in the delivery of healthcare services (12). There is clear evidence that nurses’ work engagement plays a key role in the quality of care (13). A high level of work engagement positively affects nurses’ physical and mental status and career development, stimulating their work potential, reducing their willingness to leave, and directly influencing the presentation of patient health-centered care behaviors (14).

There are many unpredictable situations that can happen to nurses in the course of their work. These situations can cause great psychological stress for nurses (15). One study showed that nurses’ positive psychological factors are positively correlated with work engagement (16). Resilience is a positive psychological factor that improves nurses’ mental health, reduces occupational stress, and enhances their productivity and intrinsic motivation (17). Another factor often emphasized in nurses’ work engagement is organizational support (18). Organizational support is defined as the respect and care that employees feel from their organization or institution (19). Many studies have determined that organizational support is associated with nurses’ work engagement (20, 21). Overall, both resilience and organizational support were positive factors that influenced nurses’ work engagement.

The innovative behaviors of nurses are those that are designed to promote health, prevent disease, and improve the quality of care for patients. The process by which nurses seek and develop new methods, techniques, and ways of working introduces and applies them to their work behaviors after obtaining the support of others (22). Research supports the idea that innovative behavior is significantly associated with work engagement (23). As an indispensable key force during medical reform and development, nurses are not compatible with the rapid development of clinical medical disciplines, and their high or low innovation abilities directly affect the overall innovation ability of the hospital.

**Figure 1:**
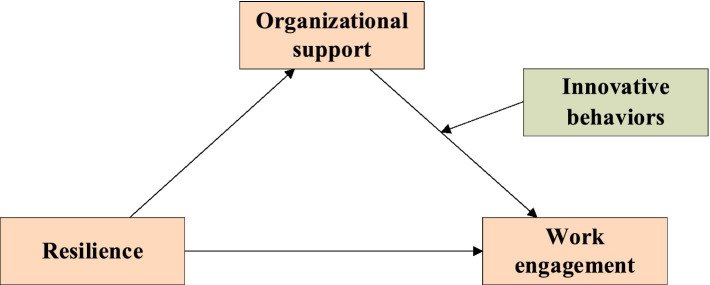
Hypothesis of this study.

Resilience, organizational support, innovative behaviors, and work engagement are all related. However, the pathways among them that influence work engagement have rarely been explored. The theoretical model we applied was the Job Demands-Resources (JD-R) model. According to the JD-R model, the inter process of job input that the gain process is triggered by an abundance of job resources, including resilience and organizational support. In addition, innovative behavior is a positive individual factor. This study proves that innovative behavior is significantly related to the innovative climate provided by an organization (organizational support) (24). Therefore, based on the JD-R model and literature review, we developed a research hypothesis as shown in Figure 1. Resilience, organizational support, and innovative behavior can synergistically influence nurses’ work engagement through a certain path.

## 2 Methods

### 2.1 Design, setting, and participants

This study employed a cross-sectional design. Participants were recruited from July 2022 to December 2022 using convenience sampling taken from four tertiary hospitals in Hunan, China. The inclusion criteria of all participants were as follows: (1) obtaining a licensed nurse qualification certificate and having more than one year of nursing experience and (2) voluntarily participating in the survey. Exclusion criteria were as follows: if participants (1) had more than 3 months of vacation in the past year and (2) had left nursing. This study was conducted using an online survey and questionnaire. The recruitment poster included the purpose of the study, criteria, procedures, and contact information for the corresponding author. The corresponding author obtained informed consent from nurses who volunteered to participate in the study and distributed the online link. The study participants completed the questionnaire through an online link.

We used the formula *n* = (Zασ/δ)2 to calculate the sample size. The parameter was α = 0.05, and the value of Zα was 1.96. The σ represents the standard deviation. According to the survey results of Chinese scholars on nurses’ work engagement, we took the value of σ as 10.83 (25). The δ represents the allowable error, and smaller values indicate greater sample size and precision. Previous research has shown that the allowable error can be set to 0.25 ~ 0.5 times the standard deviation (26). Therefore, the range of δ can be 2.71 ~ 5.42. However, in this study, to improve the precision of the results, we took the value δ to be 1. Considering a 10% sample attrition rate, the final sample size was calculated to be 495.

### 2.2 Measures

#### 2.2.1 Demographic characteristics

Sociodemographic data (gender, age, education level, marital status, fertility status, etc.) and occupational characteristics (hospital grade, job level, mode of appointment, monthly income, weekly working hours, and night shifts) were collected.

#### 2.2.2 Work engagement

The Chinese version of the Utrecht Work Engagement Scale (UWES-9) was used to assess the participants’ levels of work engagement (27). The scale includes 3 subscales of vitality (3 items), dedication (3 items), and concentration (3 items), with a total of 9 items. Each item is a 7-point Likert scale with a score ranging from 0 (*never*) to 6 (*every day*). The total scores ranged from 0 to 54. Higher scores indicate higher levels of work engagement. The Cronbach’s alpha coefficient for this scale was 0.96.

#### 2.2.3 Resilience

The Chinese version of the Psychological Resilience Questionnaire for Nurses was used to measure the level of resilience of the participants (28). The scale consisted of 4 dimensions: self-efficacy (3 items), hope (3 items), resilience (3 items), and optimism (3 items), with a total of 12 items. Each item was scored on a Likert 6-point scale with a score ranging from 1 (*strongly disagree*) to 6 (*strongly agree*). The total scores ranged from 12 to 72. Higher scores indicate higher resilience. The Cronbach’s alpha coefficient for this scale was 0.96.

#### 2.2.4 Organizational support

The Chinese version of the Nurses’ Sense of Organizational Support Scale was used to measure the participants’ levels of organizational support (29). The scale was divided into two dimensions, emotional support (10 items) and instrumental support (3 items), with a total of 13 items. Each item was scored on a Likert 5-point scale with a score ranging from 1 (*strongly disagree*) to 5 (*strongly agree*). The total scores ranged from 13 to 65. Higher scores indicate better perceived organizational support. The Cronbach’s alpha coefficient for this scale was 0.97.

#### 2.2.5 Innovative behavior

The Chinese version of the Nurse Innovative Behavior Scale was used to measure the level of organizational support of the subjects (30). The scale was divided into 3 dimensions generating ideas (3 items), obtaining support (3 items), and realizing ideas (4 items), with a total of 10 items. Each item was rated on a 5-point Likert scale, with scores ranging from 1 (*never*) to 5 (*frequently*). The total scores ranged from 10 to 50. The higher the score, the more innovative behavior at work is demonstrated. The Cronbach’s alpha coefficient for this scale was 0.96.

### 2.3 Data collection

This research was conducted using the WeChat applet SOJUMP. First, we selected 40 nurses in the Second Xiangya Hospital as the initial respondents and explained the inclusion criteria to them. Then, they invited colleagues who met the inclusion criteria to participate in the study. Participants had to answer questions sequentially during the fill-in process and not skip or miss questions. Three investigators verified and sifted the final data for subsequent statistical analysis.

### 2.4 Ethical consideration

The study was approved by the Second Xiangya Hospital of Central South University (Ethical review number: E2020142) and performed in accordance with the Declaration of Helsinki. Before the formal investigation, all investigators were trained on registration, checking the completeness of questionnaires, and ethical tenets of conducting research. In addition, all participants were informed about the purpose of this study and gave their consent. Not incentives or inducements were peovided to complete the questionnaire. After submission, the participants were informed that all the contents of the questionnaire would be anonymous and confidential and that it would only be used for academic research.

### 2.5 Data analysis

Statistical analysis were performed using SPSS 27.0 and PROCESS macros for version 4.2 (31). In addition to descriptive statistics, t-tests, ANOVA, and Spearman’s correlation analysis were used to test the correlation between variables. The PROCESS macro (Model 4) was used to test the mediating role of organizational support in psychological resilience and work engagement using a bootstrap sample of 5,000. In addition, the PROCESS macro (Model 14) was used to test the moderating role of innovative behavior between organizational support and work engagement by constructing a mediating model with moderation using a bootstrap sample of 5,000. The effect was considered significant if the 95% confidence interval (CI) did not include zero.

## 3 Results

### 3.1 Descriptive statistics and univariate analysis of demographic characteristics related to work engagement in nurses

**Figure 2:**
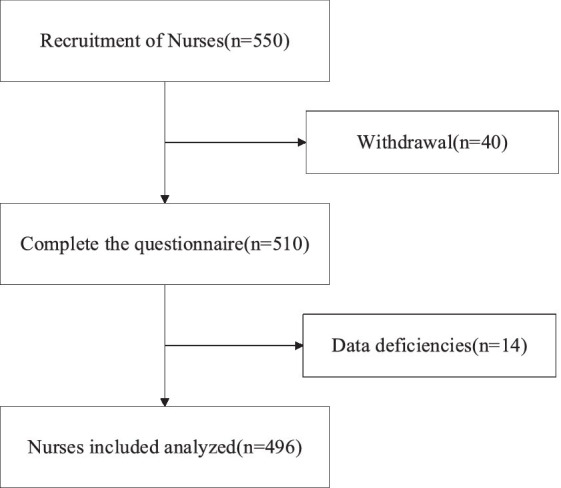
Flow diagram of participants.

As shown in Figure 2, 550 questionnaires were distributed and 510 were completed, of which 14 had missing data. Finally, 496 nurses were included. Table 1 describes the demographic and work characteristics of the participants and their relationships with work engagement. The participants were mostly women accounting for 89.3%. They were mostly from tertiary care centers. Of the participants, 74.8% were married. We distributed 550 questionnaires, and finally obtained 496 valid questionnaires, with an efficiency rate of 90.2%.

**Table 1:** Descriptive statistics and univariate analysis of demographic characteristics related to work engagement in nursess (*N* = 496).

Variables	Categories	*N* (%)	*t/F* values	*p* values
Gender	Male	53 (10.7)	t = −0.961	0.337
	Female	443 (89.3)		
Age	≤30	179 (36.1)	*F* = 7.167	<0.001
	31–40	220 (44.4)		
	41–50	79 (15.9)		
	≥51	18 (3.6)		
Hospital level	Medical centre (level 3)	436 (87.9)	*F* = 0.095	0.910
	Regional hospital (level 2)	37 (7.5)		
	Other	23 (4.6)		
Education	Junior college or below	48 (9.7)	*F* = 0.817	0.442
	College	385 (77.6)		
	Graduate school and above	63 (12.7)		
Marital ststus	Single	113 (22.8)	*F* = 0.211	0.810
	Married	371 (74.8)		
	other	12 (2.4)		
Fertility circumstance	Unfertilized	138 (27.8)	*F* = 1.937	0.145
	A child	207 (41.7)		
	Two children or above	151 (30.4)		
Professional title	Junior	180 (36.3)	*F* = 5.175	0.002
	Intermediate	242 (48.8)		
	Senior Deputy	59 (11.9)		
	Senior	15 (3.0)		
Monthly income	≤5,000	63 (12.7)	*F* = 0.937	0.392
	5,001–10,000	299 (60.3)		
	>10,000	134 (27.0)		
Weekly working hours	≤35 h	62 (12.5)	*F* = 0.458	0.712
	36-45 h	244 (49.2)		
	46-55 h	120 (24.2)		
	>55 h	70 (14.1)		
Weekly night shifts	≤1	284 (57.3)	*F* = 7.885	<0.001
	2–3	147 (29.6)		
	>3	65 (13.1)		

### 3.2 Descriptive statistics of variables

Table 2 shows the means, standard deviations, and score ranges of resilience, organizational support, innovative behavior, and work engagement. The score for clinical nurses’ work engagement was 35.61 ± 13.66.

**Table 2:** Descriptive statistics of measurements (*N* = 496).

Variables	Range	M	SD
Resilience	12 ~ 72	54.67	10.28
Organizational support	13 ~ 65	47.72	10.22
Innovative Behavior	10 ~ 50	34	7.86
Work engagement	0 ~ 54	35.61	13.66

M, mean.

SD, standard deviation.

### 3.3 Spearman’s correlation analysis of variables

Table 3 presents the results of the correlation analysis of resilience, organizational support, innovative behavior, and work engagement. Resilience, organizational support, and work engagement were positively correlated. Furthermore, innovative behavior was also positively correlated with organizational support and work engagement.

**Table 3:** Correlations between resilience, organizational support, innovative behavior, and work engagement.

	Resilience	Organizational support	Innovative Behavior	Work engagement
Resilience	1			
Organizational support	0.580^**^	1		
Innovative Behavior	0.632^**^	0.611^**^	1	
Work engagement	0.648^**^	0.622^**^	0.673^**^	1

### 3.4 Moderated mediation effect analysis

**Figure 3:**
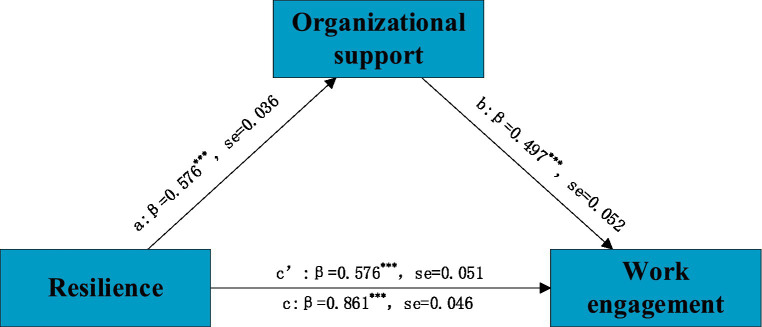
The mediated model. ***p<0.001. path c: total direct effect, path c’: direct effect, path a and b: indirect effect.

**Figure 4:**
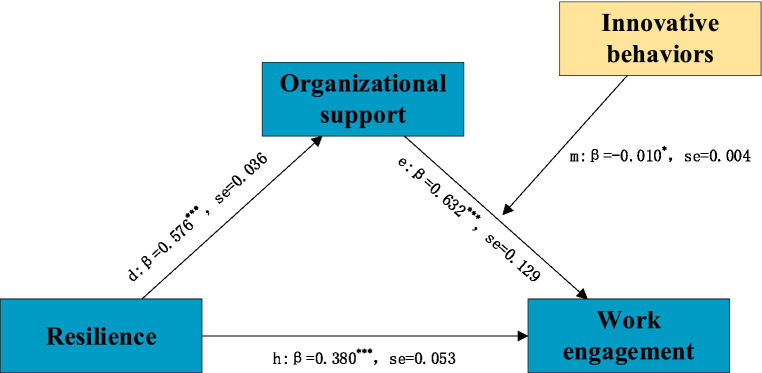
The moderated mediation model. *p<0.05, ***p<0.001. path d: relation between resilience and organizational support, path e: relation between organizational support and work engagement, path h: relation between resilience and work engagement, the moderating effect of innovative behaviors in the relationship between organizational support and work engagement.

**Figure 5:**
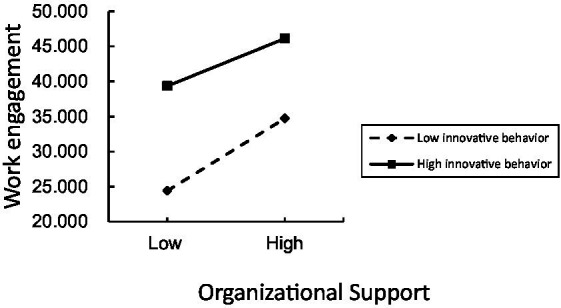
The moderation plot for innovative behavior.

Figure 3 shows that the total effect of resilience on work engagement was significantly positive (c path. *β = 0.861, p < 0.001*). Resilience was significantly and positively correlated with organizational support (a path, *β* = 0.576, *p <* 0.001; b path, *β* = 0.497). The results of the bootstrap show that the effect of resilience on work engagement is partially mediated by organizational support. According to Figure 4, the interaction between innovative behavior and organizational support could also affect nurses’ work engagement (m path, *β* = −0.010, *p* < 0.05). It demonstrates that innovative behavior plays a moderating role in the mediating path of “resilience-organizational support-work engagement.” To interpret the interaction results, we plotted the interaction effects in Figure 5.

## 4 Discussion

### 4.1 Status of work engagement among nurses

In this study, we found that nurses’ work engagement was at a moderate level. Another study in China found that nurses’ work engagement was at high levels (32). This discrepancy is likely related the differences in the participants. The latest threat to global health is the ongoing outbreak of respiratory disease, the Covid-19 outbreak, which has posed a serious challenge to the public health, research and medical communities (33). We found that nurses’ work engagement in China was lower than that reported in pre-COVID-19 Sweden (34) and post-COVID-19 Spain (35). Besides geographical differences, sociocultural differences may also contribute to the differences (36). In China, the low status and poor professional recognition of nurses for historical reasons significantly impact the enthusiasm for work. Moreover, after media coverage of doctors fighting the pandemic, the role of nurses in outbreak response was devalued, leading to decreased work engagement (37). This suggests that medical managers should understand the working environment of front-line nurses from a social perspective and formulate relevant policies to stabilize nursing teams.

### 4.2 Resilience and work engagement

We found that resilience has an important impact on nurses’ work engagement. This is consistent with previous studies (38, 39). Studies have shown hat the nursing specificity of suppressing emotions during interactions with patients and colleagues may negatively impact their work enthusiasm (40, 41). When individuals feel restricted and unable to act on their values, it may contribute to professional burnout (42). Resilience is considered a positive individual psychological resource. Some scholars have found that nurses with low levels of resilience have low professional value identity (43, 44). These nurses often fail to carry out positive self-regulation, which gradually depletes individual emotions and reduces work enthusiasm. It is believed that work engagement is based on psychological identification with work (11, 45). Work performance reflects personal core values. Nurses with high resilience can adhere to professional principles and values, and play a deeper role in their work (46, 47). They tend to demonstrate active work engagement by refracting cognition, correctly articulating ideas, and effectively managing relationships. Therefore, nursing managers should focus on improving nurses’ resilience to stimulate enthusiasm and increase work engagement.

### 4.3 Mediating role of organizational support

The JD-R model encompasses a personal resources component and has been shown to predict work engagement empirically. Organizational support is considered a positive social resource to combat the negative outcomes of negative job stress and burnout in the workplace (36, 48). Our study demonstrated that organizational support could mediate the relationship between resilience and work engagement. That is, resilience can indirectly affect nurses’ work engagement through organizational support. When nurses possess high levels of resilience, they are more likely to perceive emotional, technical support, respect and understanding from the organization, resulting in a strong sense of belonging. This sense of belonging can increase organizational dedication and promote emotional adjustment for better productivity (49). The idea of organizational personification states that the relationship between nursing managers and nurses reflects the organization’s care and support (50). This suggests that while cultivating nurses’ resilience, nursing managers should also respect and affirm nurses’ labor efforts and create a supportive work environment to increase their work enthusiasm.

### 4.4 Moderating role of innovative behavior

We found that innovative behavior plays a moderating role in the mediating path of “resilience-organizational supposition-work engagement.” Highly innovative behavior can effectively improve the effectiveness of organizational support on work engagement. This is consistent with the JD-R working model claim results (51). Individuals and the environment are mutually shaped and motivated. Individuals with great innovation abilities can mine clinical problems or opportunities, gain support from colleagues, and promote active engagement in clinical practice (52). We found that organizational support has a stronger association with job engagement than resilience, especially when innovation is taken into account. This indicates that the regulatory pathway in the model may be more critical. Furthermore, organizational support had a weaker positive effect on work engagement as innovation behavior increased (Figure 5). This suggests that interventions tailored to nurses with low levels of innovation may be more effective. Therefore, managers should encourage nurses with low innovative behavior to join nursing innovation teams and develop autonomous innovation through behavioral plans. This will promote innovation from individuals to organizations and advance practical transformation.

### 4.5 Limitations

This study has a few limitations. First, its cross-sectional design means that it is not possible to rely on the results to infer causality. Second, the population of this study was from one province in China. Therefore, the representativeness of the results may be open to examination. Further expansion of the sample size of this study is needed to verify its findings. Third, the questionnaire in this study was collected by self-reporting, which may have caused recall bias.

### 4.6 Theoretical strengths

Most previous resource studies have focused on using family resources (family mastery) (53), individual resources (autonomous decision-making, professional commitment, etc.) (51), or a combination (healthy work environment and resilience) (54) to predict work engagement, with less attention paid to social resources. In this study, we aimed to describe the impact of social resources (organizational support) and personal resources (resilience and innovative behavior) on nurses’ work engagement through the JD-R theoretical framework. Specifically, the organizational support introduced in this study can comprehensively assess nurses’ instrumental support and social support (29). According to the China Labor Force Dynamics Survey (55), the external social support of the working population is proportional to the happiness index, which is related to work enthusiasm. Moreover, Maslow’s hierarchy of needs theory also emphasizes the need for individual social support (56). Therefore, consideration of this variable not only helps to extend the JD-R model in terms of social functioning and emotion but also provides a basis for clinical development to improve nurse work engagement.

### 4.7 Practical strengths

The study showed that resilience, organizational support, and innovative behavior contribute significantly to nurses’ work engagement. Based on this evidence, it would be valuable for nursing managers to develop effective strategies based on this evidence to improve nurses’ work engagement and stabilize the nursing team, further reduce medical costs and minimize adverse health outcomes in patients. The current COVID-19 pandemic has had a significant negative impact on nurses’ work engagement. Ensuring high standards of care during and after emergencies is a top public health priority. However, these large-scale outbreaks have shown that working in stressful situations can have a significant impact on work engagement. The results of this study support the impact of psychological resilience, organizational support, and innovative behaviors on nurses’ work engagement. The findings of this study have the potential to inform supportive interventions and enhance nurses’ willingness to provide assistance in affected areas during health crises, thereby bolstering their work engagement and ultimately saving lives.

## 5 Conclusion

This study indicated that organizational support plays a mediating role in resilience and work engagement, and that innovative behaviors play a moderating role in the pathways of organizational support and work engagement. The results contribute to a deeper understanding of the pathways that influence work engagement in nurses. The results of this study suggest that policy makers and nurse managers need to focus on nurses’ resilience, improve organizational support, and develop innovative behaviors. Future studies need to further explore the networks that influence nurses’ work engagement. The intervening factors identified in this study also need to be validated by further randomized controlled trials.

## Data availability statement

The raw data supporting the conclusions of this article will be made available by the authors, without undue reservation.

## Ethics statement

The studies involving humans were approved by the Ethics Committee of Xiangya School of Nursing, Central South University (E2022142). The studies were conducted in accordance with the local legislation and institutional requirements. The participants provided their written informed consent to participate in this study.

## Author contributions

FZ: Conceptualization, Data curation, Investigation, Methodology, Software, Visualization, Writing – original draft. KL: Conceptualization, Data curation, Investigation, Methodology, Software, Visualization, Writing – original draft. HS: Data curation, Formal analysis, Investigation, Writing – review & editing.

ZY: Data curation, Visualization, Writing – review & editing. TY: Data curation, Visualization, Writing – review & editing. LD: Data curation, Visualization, Writing – review & editing. JZ: Supervision, Validation, Writing – review & editing.
